# Beauty and Uncertainty as Transformative Factors: A Free Energy Principle Account of Aesthetic Diagnosis and Intervention in Gestalt Psychotherapy

**DOI:** 10.3389/fnhum.2022.906188

**Published:** 2022-07-13

**Authors:** Pietro Sarasso, Gianni Francesetti, Jan Roubal, Michela Gecele, Irene Ronga, Marco Neppi-Modona, Katiuscia Sacco

**Affiliations:** ^1^BraIn Plasticity and Behaviour Changes Research Group, Department of Psychology, University of Turin, Turin, Italy; ^2^International Institute for Gestalt Therapy and Psychopathology, Turin Center for Gestalt Therapy, Turin, Italy; ^3^Psychotherapy Training Gestalt Studia, Training in Psychotherapy Integration, Center for Psychotherapy Research in Brno, Masaryk University, Brno, Czechia

**Keywords:** neuroaesthetics, gestalt therapy, predictive coding, field theory, psychopathology

## Abstract

Drawing from field theory, Gestalt therapy conceives psychological suffering and psychotherapy as two intentional field phenomena, where unprocessed and chaotic experiences seek the opportunity to emerge and be assimilated through the contact between the patient and the therapist (i.e., the intentionality of contacting). This therapeutic approach is based on the therapist’s aesthetic experience of his/her embodied presence in the flow of the healing process because (1) the perception of beauty can provide the therapist with feedback on the assimilation of unprocessed experiences; (2) the therapist’s attentional focus on intrinsic aesthetic diagnostic criteria can facilitate the modification of rigid psychopathological fields by supporting the openness to novel experiences. The aim of the present manuscript is to review recent evidence from psychophysiology, neuroaesthetic research, and neurocomputational models of cognition, such as the free energy principle (FEP), which support the notion of the therapeutic potential of aesthetic sensibility in Gestalt psychotherapy. Drawing from neuroimaging data, psychophysiology and recent neurocognitive accounts of aesthetic perception, we propose a novel interpretation of the sense of beauty as a self-generated reward motivating us to assimilate an ever-greater spectrum of sensory and affective states in our predictive representation of ourselves and the world and supporting the intentionality of contact. Expecting beauty, in the psychotherapeutic encounter, can help therapists tolerate uncertainty avoiding impulsive behaviours and to stay tuned to the process of change.

Question: *Would it be correct to suggest that the aesthetic is this unifying glimpse that makes us aware of the unity of things which is not (in the limited sphere of) consciousness?*

Gregory Bateson: *That is right; that is what I am getting at. The flash which appears in consciousness as a disturbance of consciousness is the thing that I am talking about.*

*(Bateson, 1991; p. 300)*

## Introduction

We are instinctively drawn to seek causal relations in the sensory regularities we observe. Not only natural sciences support this view, but also a number of philosophers and poets: e.g., Kant considers causality as an *a priori* concept, and Goethe affirms that cause and effect are the “most innate concepts” (Heiddeger and Boss, 2000; p. 230). However, as suggested by modern developments in quantum physics (Heisenberg, 1927; Schrödinger, 1944; Fantappiè, 2011) explaining phenomena uniquely by the causality principle might be scarcely effective in natural science and, as widely discussed by Heidegger in the Zollikon Seminars, completely misleading and objectifying when approaching human suffering and healing (Meynen and Verburgt, 2009). Indeed, suffering is not only “caused by” but it has an intention, it tends towards something (Spagnuolo Lobb, 2013; Roubal et al., 2017; Bloom, 2020). Even severe psychopathological suffering, after all, makes sense and “is about something,” i.e., it carries (to suffer: from latin *sub-ferre*, to carry) an intentionality for reaching out and making sense of the world (i.e., intentionality of contacting in Gestalt theory; Francesetti, 2015, 2021; Bloom, 2020). As any self-regulating autopoietic biological system (Maturana and Varela, 1980), humans are intrinsically oriented to growth (Dempster, 2000). When possible, as we will better discuss in the third section, self-regulating systems disrupt their habitual policies (Kiverstein et al., 2019; Seth et al., 2020; Van de Cruys et al., 2020) to integrate and create new dynamics (Kelso, 1995; Miller et al., 2021). Both being and becoming at once, even when they suffer, “people are inherently self-regulating and growth-oriented and […] their behaviour, including symptoms, cannot be understood apart from their environment” (Yontef and Jacobs, 2005). Gestalt therapy approaches rest on this fundamental assumption.

If suffering has a meaning and is oriented toward a relational “next” (Spagnuolo Lobb, 2013), the main question in giving support is “where is the person headed to?” (Roubal et al., 2017). In other words, in the gestalt approach the therapist “trusts organismic self-regulation more than therapist-directed change attempts” and is concerned with creating the conditions “that focus attention where needed for healing and growth” (Woldt and Toman, 2005; p. 82). The attentional attitude of the therapist and his/her ability to tolerate uncertainty (Staemmler, 1997, 2000, 2006) is thus essential: “To be aware, awake, with senses active, and at the same time relaxed, allowing you to be touched by what happens. To remain confident that chaos does indeed make “sense,” and that with sufficient support a meaning will emerge. The therapists are ready to gather intentionality and to support its unfolding. It is the intentionality towards contact that brings order to intersubjective chaos” (Roubal et al., 2017; p. 6). Suffering represents an opportunity for the patients to get in contact with their unintegrated or chaotic experiences: when possible, its causes should not be eliminated but rather supported in their intentionality. As we will further explain in the second section, contrarily to the medical approach, Gestalt therapists do not aim at reducing the pain of the patients but at sharing it with them, in order to help them process experiences that couldn’t be processed and assimilated without the supportive presence of the other (Francesetti, 2019a,b).

Within this theoretical framework, the aesthetic sensibility of the therapist is a key factor for the success of a therapeutic intervention (Bloom, 2011; Francesetti, 2012; Spagnuolo Lobb, 2018). Gestalt therapy might indeed be considered a fully-fledged evaluative process (Perls et al., 1951) following intrinsic aesthetic criteria (Francesetti and Gecele, 2009; Bloom, 2011): “the achievement of a strong Gestalt is itself the cure” (Perls et al., 1951, p. 232). According to Gestalt therapy, when assuming an aesthetic attitude, also referred to as aesthetic diagnosis (Roubal et al., 2017), the therapist can tolerate sensory, emotional, and relational uncertainty (Francesetti, 2019b) without avoiding it, thereby providing the necessary support for change (Spagnuolo Lobb, 2018). In the words of Wilfred Bion: “beauty makes a very difficult situation tolerable” (Lord, 2017). The aesthetic diagnosis (from the Greek *diagnosis*, meaning to know through; Cortelazzo and Zolli, 1983) is *per se* a transformative experience, with the potential of being therapeutical: it may defy common knowledge-acquisition processes oriented to predictability and control, while re-orienting the physiological arousal to learning and change (Sarasso et al., 2020a). A crucial starting point for the therapist to trigger this process is to gather raw sensory impressions without categorising them in predefined knowledge representation schemes: transient states of not-knowing with increasing tension of stimulus may later make such sensory impressions thinkable, as already intuited by psychoanalysis (Freud, 1951). Bion suggested that the only way for someone to stay on the path to cure is through the tolerance of doubt and intimacy with the unknown (Bion, 1994). Bion, indeed, similarly to Gestalt therapy, encouraged the therapist to completely surrender to the therapeutic process, he even discouraged therapists from maintaining a desire to cure patients (Aguayo and Malin, 2013).

Such holistic perspective, also referred to as the paradoxical theory of change (Beisser, 1970; Francesetti and Roubal, 2020) will be discussed within the neurocomputational framework of cognition called Free Energy Principle and its neural implementation called predictive coding (PC), which has recently gathered a wide consensus among neuroscientists (Friston, 2010) and is starting to inform psychopathology (Barrett et al., 2016; Badcock et al., 2017, 2019; Ciompi and Tschacher, 2021; Smith et al., 2021) and clinical practice (Tschacher et al., 2017; Holmes and Nolte, 2019). The basic principle of FEP and PC (Section “Experimental Evidence Linking Aesthetic Pleasure and Learning”) is that agents constantly update the predictive representation (Sims and Pezzulo, 2021) of their environment based on Bayesian inference drawn from unpredicted sensory input while inhibiting uninformative predicted input under the imperative of minimising variational free energy (i.e., uncertainty; Friston and Kiebel, 2009; den Ouden et al., 2012). The FEP resonates with Gestalt therapy description of the organism-environment interaction (see Section “Experimental Evidence Linking Aesthetic Pleasure and Learning”), and with the principles of Gestalt psychology (Van de Cruys and Wagemans, 2011b). Indeed, Gestalts can be defined as inferred predictive models of the environment (Van de Cruys and Wagemans, 2011a), and, mirrorwise, predictive inferences behave as Gestalts: “in the predictive coding loop, the inferred cause (the idea, the whole) predicts the evidence, while, at the same time, the evidence (the words, the parts) modifies the inferred cause” (Frith and Wentzer, 2013; p. 658). As we will discuss in Section “A Bridge Between Gestalt Field Theory and the Free-Energy Principle,” the FEP might provide a valid theoretical framework to interpret neurophysiological data to inform evidence-based psychotherapy. Coherently with previous accounts, our main take is that psychotherapy “stimulates bayesian inference, enabling experience and feeling states to be “metabolised” and assimilated […] without being overwhelmed by psychic entropy” (Holmes and Nolte, 2019; p. 1).

Furthermore, the FEP is vitalistic, in the sense that it postulates the existence of an intrinsic intentionality of living systems (i.e., free energy minimisation; Bruineberg and Rietveld, 2014), which could be considered the formal biological basis for the emergence of the representational intentionality or “aboutness” in living systems (Ramstead et al., 2020; Wiese and Friston, 2021), as well as for skilled intentionality (Bruineberg and Rietveld, 2014; Kiverstein et al., 2020), i.e., the organism tendency towards an optimal grip on a field of affordances (Merleau-Ponty, 2002). In Section “A Bridge Between Gestalt Field Theory and the Free-Energy Principle,” we will further discuss how the systematic tension toward free energy minimisation emerges in complex systems encompassing more than one organism, as in the case of the therapeutic situation. Indeed, the FEP is a theory focused on the functioning of the boundary between the organism and the environment, which does not take subjectivity for granted. On the contrary, the FEP describes the inferential nature of the cognitive processes underlying our sense of being a self separated from the external environment. For this reason, FEP intuitions might support the current transversal paradigmatic shift beyond relational approaches toward field theory in psychotherapy (Francesetti, 2019b), a novel approach that radically that takes into account the pre-subjective continuous and indissoluble interaction between the organism and the environment (Francesetti and Roubal, 2020).

Furthermore, in Section “Aesthetics and Knowledge/Change,” we aim at reviewing and discussing the experimental, theoretical and neurocomputational evidence derived from experimental aesthetics, neuroaesthetics and psychopharmacology suggesting that: (1) aesthetics sensibility might have evolved as an hedonic feedback from the process of growth at the contact boundary between the organism and the environment; (2) beauty perception motivates us to momentarily tolerate uncertainty to change our internal representation of the world and of our relation with the latter (Sarasso et al., 2020a). Finally, we will discuss how aesthetic sensibility could support therapeutic change in the clinical setting. Current developments in neuroaesthetics have indeed renewed the interest in the link between knowledge/meaning and beauty (Sarasso et al., 2020a, 2021c), perhaps supporting the hypothesis that aesthetic sensibility and competences are key factors for the success of the therapeutic encounter.

Lastly, we included a brief glossary in Appendix A, should the reader need additional information on specific terms.

## Suffering of Experience: Gestalt-Phenomenological Approach to Psychopathological Fields

### The Field Perspective: A Dive Into the Undifferentiated

The dominant medical approach in clinical psychotherapy and psychiatry today makes use of abstract third-person descriptive diagnosis and clinical protocols aiming at changing the way the patient functions (Barron, 1998; Francesetti and Gecele, 2009). The risk of this approach is that the connections between the symptoms and the environment fails to be grasped and the transformative potential of suffering is overlooked or silenced (Francesetti, 2019a). Apart from being problematic on a clinical level (Bracken et al., 2012), this dominant clinical approach possibly reflects a fundamentally erroneous ontological conception of human nature, perhaps initiated with Descartes (Damasio, 1994; Heiddeger and Boss, 2000): “a disconnection, from the continuity with the original unitary field [*between the psychic and the social environment*] (Perls et al., 1951, p. 271).” We propose that field theory (Latner, 1983; Parlet, 1991; Yontef and Jacobs, 2005; Spagnuolo Lobb, 2013; Philippson, 2017; Francesetti and Roubal, 2020; Bloom, 2021) is currently a most promising attempt to overcome these shortcomings.

We all have a natural experience of ourselves as selves separated from the environment upon which we can act. Perls et al. (1951, p. 263) define this mode of experience: “an unavoidable illusion, empirically given in average experience,” while Husserl calls it the “natural attitude” (Husserl, 2012), where objects are something given out there that I can perceive as separate from me. However, along with selfhood, the Self and the Other are incessant and unending processes emerging from an undifferentiated ground (Husserl, 2012), where they are not yet defined (Perls et al., 1951; Philippson, 2009; Bloom, 2019; Francesetti and Roubal, 2020). Subjects are not given, but constantly emerge as an expression of the situation (Philippson, 2009; Francesetti, 2019b) that precedes them and that provides a constitutive momentum. From a neuroscientific perspective, according to Damasio, the first step of this process is the proto-self, a stage where perception is not defined as mine yet; it becomes my perception only in the second stage, that of the subjective-self (Damasio, 2012). Metzger (1941) referred to this second-stage outcome as the *Endgestlat*, which emerges from a diffuse, undifferentiated, and global initial perceptual moment called *Vorgestalten*.

As we will further discuss, recent neurocomputational accounts of cognition confirm the pre-subjective root of perception and behaviour. Similarly to the phenomenological perspective (Merleau-Ponty, 2002; Waldenfels, 2011; Husserl, 2012; Alvim Botelho, 2016) and early Gestalt psychology intuitions [see as an example the work by Metzger (1941)], current neuroscientific perspectives see the representation of subjects as separated from stable objects as the outcome of an inferential perceptual process occurring at the “statistical” boundary (i.e., the Markov blanket, see Section “Predictive Coding Accounts of Aesthetic Appreciation”) between the organism, or a system of sensorily coupled organisms, and the environment (Friston, 2010; Hohwy, 2016). These findings suggest that clinical theory and practice should overcome the natural experience of the subject-object split and investigate and take into account pre-subjective field dynamics. Indeed, different theories across a wide variety of clinical approaches (Ogden, 2003; Ferro and Civitavese, 2016; Bourguignon and Katz-Gilbert, 2018; Francesetti and Roubal, 2020) are starting to recognise that in the complex and chaotic (Francesetti, 2019a) psychotherapeutic field or situation, the forces and tensions that move the therapist and the patient belong to the field or situation itself. These forces of the field (Lewin, 2016) are intentionalities: intrinsic tensions moving towards the fulfilment of the potentialities of the situation (Francesetti and Roubal, 2020). In metaphorical terms: “The forces belong to the situation—it is not just about the client and it is not just about the therapist. What emerges is different from the sum of the parts, in much the same way as when a molecule of oxygen and two molecules of hydrogen meet and a new, unique quality of water appears.” The group or dyad behaves as a whole (Bion, 1961). The relationship between parts and whole is one of mutual constraints which Tschacher and Haken defined as circular causality (Tschacher and Haken, 2007). That is why in the patient-therapist interaction, the priority is not to distinguish “what is mine” from “what is yours,” what matters is to recognise the forces that provide momentum to subjects and let them transform the field (Francesetti and Roubal, 2020).

Field forces push towards the kind of contact where the potentialities of the field can be developed, where the situation dynamics can be transformed through the assimilation of novel experiences (Francesetti and Roubal, 2020). We hypothesise that field intentionalities correspond to the tension aiming at reducing entropy central to current accounts of perception, cognition, and action (Friston, 2010; Clark, 2013; Hohwy, 2016). Entropy in a self-regulating lively system can be reduced either through active sampling of sensory inputs, i.e., acting according to previously acquired behavioural and perceptual patterns, thereby leading the field to a rigid given set of states, or, alternatively, through the update of the predictive representation of the sensory causes of stimuli. As we will see in Section “A Bridge Between Gestalt Field Theory and the Free-Energy Principle,” the tension to minimise entropy becomes shared as soon as two organisms synchronise: it becomes a tension of the field. Change must thus necessarily go through a (aesthetic) dive into the shared pre-subjective perceptual milieu.

### Psychopathology: Growing With Unprocessed Experiences

Without the pretence of being exhaustive, for the purpose of the present paper, we will consider suffering not as an attribute of an individual, but rather, as an emergent property (Francesetti, 2019a) of the field in the therapeutic situation. In these terms, suffering is the result of a rigidity of experiential possibilities (Francesetti, 2019b). Perception, cognition, or emotion are dulled or restricted and embodied affective resonance (Fuchs and Koch, 2014) is constrained by the limits intrinsic to the psychopathological situation itself. This limited set of possible and probable experiences becomes shared as soon as the patient and therapist meet. They both suffer. Indeed, in the therapeutic session, the psychopathological field emerges as a sort of landscape already inhabited by the patient and therapist, that moves them (Pallaro, 2002; Fuchs and Koch, 2014) and can be experienced as an atmosphere (Francesetti, 2019a). Although psychopathological atmospheres ontologically do not exist as “external objects,” these *almost-entities* unfold between and around subjects; they are actualised in complex systems and permeate feelings, bodies, languages, narrations, and cultures (Francesetti, 2015). Becoming aware of our somatosensory and affective resonances within psychopathological atmosphere is crucial in Gestalt therapy (Francesetti, 2019a). Psychoanalysts (Shaw, 2003), also see the therapist’s body as a vital element of the therapeutic encounter and may refer to this awareness of how we are “moving through and being moved” (Boston Change Process Study Group, 2018) as somatic countertransference (Margarian, 2014): “the therapist’s awareness of their own body, of sensations, images, impulses, and feelings that offer a link to the client’s healing process” (Orbach and Carroll, 2006, p. 64).

The etiopathogenesis of psychopathology is related to functional adjustments (Zinker, 1977) that evolved to adapt the organism or a system of organisms, such as a family or community, to a difficult situation (Francesetti, 2015). Psychopathological fields are the result of our ability to creatively adjust to what could not be fully experienced processed and assimilated, because the organism-environment field lacked the necessary social support. When support from others is absent, anesthetised feelings (typically solitude, sadness, anger, pain, and terror) cannot be assimilated and remain as more or less chaotic and disorganised sensorial footprints (Francesetti and Roubal, 2020). Affects are behavioural heuristics that allow us to respond quickly and automatically to environmental threats and opportunities (Damasio, 1996), while the conscious experience of feelings permits us to change future plans and expectations on ourselves and the world (Panksepp, 1998, 2010; Damasio, 2010), allowing for more flexible and effective corrective measures than neural mapping of body-states alone (Damasio and Carvalho, 2013). Not all affects however are able to become proper feelings when social support is missing. Even at a neurophysiological level, the presence of others can make us more or less attuned to sensory and affective uncertainty (Sarasso et al., 2022). For example, John Bowlby described affective detachment or denial as the last defensive phase of early childhood abandonment (Bowlby, 1960). The detachment system is activated when the attachment figure remains unavailable for too long and grief and mourning become unbearable; as a result, a psychopathological field becomes structured around the necessity to avoid feeling too much pain (Francesetti and Griffero, 2019).

We have the ability to encapsulate unprocessed experiences (somewhere else referred to as “non-represented bits of experience”; Botella and Botella, 2004; Levine et al., 2013; Lord, 2017), in order to make them as less disturbing as possible but this can result in the anesthetised dissociation between affective states and the experience of an emotion (Francesetti and Roubal, 2020). As an example, “peritraumatic dissociation” (Marmar et al., 1994; van der Hart et al., 2008; Danböck et al., 2021), could be considered as an outcome of such defensive self-anaesthesia mechanisms driven by the autonomic system activation to protect the organism (van der Hart, 2021).

Unprocessed experiences, however, do not simply disappear, but remain inscribed in the experiential possibilities of a given field (Francesetti et al., 2020). Meanwhile, our habitudinal behaviour, mood, and personality becomes structured around the avoidance of certain experiences (Francesetti et al., 2020). Sensorial footprints or proto-feelings continue to affect the body, to influence and stimulate perception and behavioural reactivity along repetitive patterns. When we are immersed in a repetitive experiential field, either as a patient or as a therapist, we don’t know the cause of our sensations and behaviours, but still, we sense something is happening to us (Francesetti, 2019a). Indeed, what is not assimilated and transformed nevertheless emerges in the therapy situation, together with the potentiality for its transformation, i.e., the potentialities of taking a form. The more the proto-feelings are unformulated, unspeakable and dissociated, the more they push throughout our body to be actualised and appear as something disturbing (*Unheimlich* in Freud’s terms). When entering psychopathological fields, as described by early gestalt psychologists, we remain occupied by unfinished Gestalts (*Zeingarnik effect*; Denmark, 2010) and experience intrusions by Gestalts that are not concluded (*Ovsiankina effect*; Oyama et al., 2018).

### Natura Sanat: The Role of Aesthetic Attitude in Allowing Change

Nature, when allowed to, heals itself through change. In the therapeutic field, change is achieved by changing the therapist’s attitude: how she or he is with the client. It is the way therapists are bodily present with the client that is changed in the healing process. It is only when we are not trying to change the client that the dissociated proto-feelings can be embodied and emerge (Roubal and Francesetti, 2022). It is the field intentionality of contacting that provides the fundamental momentum for change in therapy and constantly pushes for proto-feelings to become feelings (Roubal and Francesetti, 2022). As we will hypothesise in Section “I Know I Don’t Know: Paradoxical Strive for Uncertainty,” this might be due to the fact that humans are equipped with second order expectationsto assimilate ever greater levels of uncertainty into their predictive models of the self and the environment. This section will discuss how “beauty can supports us” (Francesetti, 2012; p. 10) in “knowing through feelings” (Francesetti, 2012; p. 5).

To accomplish this task, we also have a natural system to assimilate novel experiences beyond repetitive experiential patterns, thereby fulfilling field potentialities when possible. The contact intentionality of the field, the tension to bring order into intersubjective chaos (see Section “Merging Predictions in the Pre-subjective Chaos”), urges us to feel and experience more intensely. It is precisely at that moment that the therapist is “lending her flesh” (Francesetti, 2019b) to the field’s forces so that what was dismissed and left unformulated can become a figural Gestalt. In that precise moment, the therapist is seized by a proto-feeling that needs his or her body open to experience it (Francesetti, 2019b). The fundamental working hypothesis of the present paper is that the evolutionary tool that allows us to “turn up the volume” of proto-feelings and dismantle the repetitive organisation of an experiential field corresponds to our aesthetic sensibility [we called this function the “aesthetic valve” in a previous paper, see Sarasso et al. (2020a)]. Indeed, Gestalt therapy sees aesthetic sensibility as a fundamental diagnostic tool signalling to the clinician the suitability of the direction of therapy (Francesetti and Gecele, 2009; Spagnuolo Lobb, 2013), i.e., whether the couple is moving toward the assimilation of proto-feelings. If the extrinsic diagnosis is a sort of map that orients the therapist in different psychopathological experiential landscapes, the intrinsic aesthetic diagnosis provides a sense of direction. When the structure of the field changes and novelty is assimilated, we experience beauty (Francesetti, 2012). As in Gregory Bateson’s aesthetics, beauty and ugliness are related to the incompleteness of a self-organising system structure. Ugliness is a case of pathogenic blockage or confusion between an information and the total system that is its overall context (Harries-Jones, 2004).

Even before the beauty-feedback signal is sensed, the expectation of beauty in the therapeutic encounter can motivate the therapist to remain open to embody unformulated intersubjective chaotic proto-experiences. The expectation of beauty (we previously called it aesthetic attitude; Stolnitz, 1978; Sarasso et al., 2020a) might sustain the field intentionality to assimilate ever deeper layers of sensory chaos. Similarly, Jungian psychotherapists consider the aesthetic attitude as part of what Jung called the transcendent function to create new symbolic possibilities for the growth of consciousness (Beebe, 2010). When assuming an aesthetic attitude, we can better tame our instinctive reactions which are functions of the forces organising the psychopathological field. We actively inhibit our tendencies to change the situation, we do nothing and allow whatever is happening to us. In this way we may welcome what was dissociated and remained unformulated. In this moment of full acceptance of our feelings, the change has already started to happen. On the contrary, as we will further discuss in Section “Beauty Makes Us Curious (and Less Anxious)”: “When the situation has too much novelty for us and we are not self-supported enough as therapists, we start to be anxious. It is because we do not know well enough who we are in that situation, so we cannot lose ourselves for the moment. The personality function is not supporting us enough to be taken by whatever comes. As a result, we start to take care of ourselves forgetting the client for the moment. Our interventions—which seem to be made to help the client—are in fact helping to calm down our own anxiety” (Roubal and Francesetti, 2022).

## Aesthetics and Knowledge/Change

### Review of Theories Linking Aesthetics and Knowledge Acquisition: From Aristotle to Neuroaesthetics

Some recent aesthetics and neuroaesthetics theories consider the aesthetic experience as a knowledge experience (Consoli, 2015). This interpretation is not new: starting with Aristotle and throughout the Western thought, aesthetic emotions have been related to knowledge and meaning-making. In Aristotle’s Poetics, the philosopher affirms: “to be learning something is the greatest of pleasure […] The reason in delight in seeing a picture is that one is at the same time learning-gathering the meaning of things” (Tracy, 1946, p. 43). Modern aesthetics itself as a discipline has seen its rise following the romantic re-evaluation of the senses and corporality in the study of knowledge acquisition processes (Gross, 2002). German philosopher Baumgarten (1735) first defined aesthetics (*epistêmê aisthetikê*, i.e., aesthetics, the science of what is sensed) as “the science of sensory knowledge directed toward beauty” (Berleant, 2015, p. 1). Baumgarten’s aesthetics was later extended to the interpretation of sensory experience by Kant, who first linked aesthetic emotions with the correspondence between mental models and the world (Perlovsky, 2010). Schopenhauer further developed this concept and claimed that during aesthetic experiences the beholder becomes a “pure will-less, painless, timeless subject of knowledge” (Schopenhauer, 1969; p. 179).

Philosopher of art Monroe Beardsley (1981) first explicitly described aesthetic appreciation in terms of a cognitive process. He suggested that aesthetic experiences occur when attention is firmly focused on the perceptual features of the object (Marković, 2012). Similarly, according to the philosopher Dewey, aesthetic experiences maintain the attentional focus of the perceivers on the ever-changing present moment and thus prevent the engagement in routinely and mechanical interactions with the environment (Stroud, 2010). Dewey proposed that during “transformative aesthetic experiences,” perception is fully receptive, and it replaces the mere recognition of objects (Girod and Wong, 2005), including other individuals (Pappas, 2008).

More recently, drawing from experimental evidence, Menninghaus and colleagues proposed the Distancing-Embracing model (Menninghaus et al., 2017): during aesthetic experiences, the transient suspension of prototypical motor responses, resulting from perceivers’ absence of personal goals and environmental threats, makes room for a higher intensity of the felt sensations and emotions. “Moving” gives way to “being moved” (Fuchs and Koch, 2014; Menninghaus et al., 2015; Vuoskoski and Eerola, 2017). Such “aesthetic presence” enables observers to direct attention to perception for its own sake, with the subjectively felt intensity of present sensation and emotions being a reward on its own right (Menninghaus et al., 2019). Aesthetic experiences might thus be characterised by the shift of attentional deployment toward sensory input perceptual features which overcomes the automatic motor programming driven by semantic stimulus contents (Cupchik et al., 2009; Sarasso et al., 2020a). This is how Tellegen and Atkinson (1974) defined *absorption:* episodes of amplified attention that fully engage the subject’s perceptual resources and lead to self-altering experiences (Marković, 2012). As we discussed in Section “Natura Sanat: The Role of Aesthetic Attitude in Allowing Change,” aesthetic perception might not be concerned with the extrinsic homeostatic and utilitarian value of stimuli, but rather with the intrinsic quality (i.e., informational value) of the aesthetic object itself (Marković, 2012). Neuroaesthetics describes this aspect of aesthetic perception as disinterested interest and relates it to the activation of the “liking-without wanting” neural network (Chatterjee and Vartanian, 2014; Berridge and Kringelbach, 2015; Sarasso et al., 2020a).

Similary, Gallese’s “liberated embodied simulation” theory proposes that aesthetic appreciation may be induced by enhanced mirror system activation (Gallese, 2017, 2018), underlying the empathic resonance with the emotional content of works of art and interpersonal communication (Freedberg and Gallese, 2007; Gallese and Sinigaglia, 2011). Embodied simulation, which the author hypothesises to be central in psychotherapy as well (Gallese et al., 2007), is “liberated” during aesthetic experiences, in the sense that it is free from threats and urgencies (Gallese, 2018). The potentiation of mirror mechanisms may be obtained *via* the inhibition of motor responses: “immobility, that is, a greater degree of motor inhibition, probably allows us to allocate more neural resources, intensifying the activation of bodily-formatted representations, and in so doing, making us adhere more intensely to what we are simulating” (Gallese, 2017, p. 48). Indeed, the primary motor cortex was shown to be more strongly activated when participants viewed paintings (Kawabata and Zeki, 2004) or sculptures (Di Dio et al., 2007) they rated as ugly. The correlation between motor inhibition and aesthetic appreciation of musical sounds was also found in the auditory domain by Sarasso et al. (2019).

From a more “cognitive” standpoint, other researchers who studied insight phenomena have claimed that creation and manipulation of sense/meaning itself should be rewarding (Ramachandran and Hirstein, 1999). Having an insight into perceptual Gestalts *per se* might be sufficient to trigger aesthetic pleasure (Muth and Carbon, 2013). Since Gestalts can be seen as predictive representations of the environment (Van de Cruys and Wagemans, 2011a), as we will further explore in Section “Experimental Evidence Linking Aesthetic Pleasure and Learning,” the link between insight (i.e., the formation of a new Gestalt) and beauty perception is central for our purpose of bridging the gap between Gestalt accounts of aesthetics in psychotherapy and neuroaesthetics models based on the FEP.

According to Schoeller and Perlovsky (2016) and Perlovsky and Schoeller (2019) aesthetic emotions are related to the satisfaction or dissatisfaction of the so-called Knowledge Instinct, i.e., the drive to acquire knowledge about the external and internal world and perceive events as meaningful (Perlovsky, 2010). Beauty is perceived when the everlasting refinement of the mental representation of the world reduces the overall dissonance of the cognitive system (Perlovsky, 2010). The formal theory of aesthetics proposed by Schmidhuber (2009) similarly posits that the subjective aesthetic value of a stimulus depends on previously stored knowledge and the steepness of the learning curve (Schmidhuber, 2009). Altogether, as we will further explore in Section “Experimental Evidence Linking Aesthetic Pleasure and Learning” recent computational accounts of aesthetics suggest that liking is a function of the subjective process of going from a state of high uncertainty to a state of lower uncertainty (Van de Cruys and Wagemans, 2011b; Van de Cruys, 2017).

### Experimental Evidence Linking Aesthetic Pleasure and Learning

#### Gestalt Effects, Insight, and Semantic Priming

Having an insight into images (the so-called “aha moment”; Friston et al., 2017; Van de Cruys et al., 2021) can be conceptualised as a form of intrinsic reward causing the appreciation of visual images and fostering memory formation (Van de Cruys et al., 2021). Muth and Carbon (2013) demonstrated that liking significantly increased after having an insight for a Mooney image. Similarly in a previous study the authors found a strong relationship between the detectability of objects (the formation of a clear Gestalt) in cubist paintings and likings (Muth et al., 2013). This suggests that information gains reducing the uncertainty associated to sensory inputs are at the core of aesthetic experiences. When, starting from entropic and ambiguous sensations, sensory inputs are well-explained by the perceived gestalts, our brain generates an hedonic feedback (Van de Cruys et al., 2021). The disambiguation of uncertain stimuli might also result in the subjective experience of processing fluency (Graf and Landwehr, 2017). Graf and Landwehr’s Pleasure-Interest Model of Aesthetic Liking (Graf and Landwehr, 2015) assumes that the experience of fluent perceptual processing triggers aesthetic pleasure while the reduction of disfluency elicits aesthetic interest. Insight into a Gestalt corresponds to a processing fluency gain which is known to be associated to positive affect (Topolinski and Reber, 2010). The link between likings and meaning-making might also explain why a meaningful prime (reducing uncertainty high up in the cognitive hierarchy) makes the subsequent aesthetic experience more pleasurable than an incoherent Chomsky prime (a sentence devoid of meaning; Schoeller and Perlovsky, 2016).

#### Simulation Theories and Motor Priming

Meaning-making, however, is not a prerogative of sensory and cognitive disembodied processes. The motor system is not just a mere movement controller, but an integral part of our cognitive system (Gallese and Lakoff, 2005). In simple terms, we “know” also through motor simulation. As demonstrated by mirror neuron research (Rozzi et al., 2006; Rizzolatti and Fogassi, 2014), we make sense of our experience also through motor resonance encoded in the activation of subset of cross-modal sensorimotor neurons (Gallese et al., 1996; Freedberg and Gallese, 2007; Keysers et al., 2010). We understand others and objects’ affordances because we possess an internal motor representation of what we observe (Umiltà et al., 2001; Rizzolatti and Sinigaglia, 2010). Such “embodied simulation” is also essentially implicated in aesthetic experiences (Freedberg and Gallese, 2007; Cross et al., 2011; Gallese, 2017). Motor simulation underpins an empathic response contributing to aesthetic appreciation (Ticini et al., 2015; Kirsch et al., 2016). In line with this view, during the observation of paintings as compared to modified, non-artistic stimuli, transcranial magnetic stimulation (Battaglia et al., 2011), fMRI (Lutz et al., 2013), and EEG (Umilta’ et al., 2012; Sbriscia-Fioretti et al., 2013) studies revealed greater activation of fronto-parietal areas, known to match motoric models of action execution with action observation (Rizzolatti and Craighero, 2004). Interestingly, it seems that the strength of motor resonances and the ease of the simulation correlate with aesthetic appreciation. As an example, it has been shown that mimicking the emotional expression depicted in classic portraits increases their liking, in those individuals reporting higher disposition to identify with others (Ardizzi et al., 2020; Finisguerra et al., 2021). Furthermore, Leder et al. (2012) demonstrated that participants’ aesthetic appreciation of canvases was enhanced after they performed actions that matched an artist’s painting style; even the observation of static images depicting the actions corresponding to painting styles (e.g., pointillist-style dabbing of paint) produces the same effect. Indeed, the aesthetic appreciation of paintings is enhanced by priming canvases with photos of actions that match the artist’s painting style (Ticini et al., 2014). Altogether, this evidence suggests a possible link between motoric simulation, empathy, and the observer’s aesthetic appreciation (Gernot et al., 2018). In sum, an insightful understanding of what we observe is strongly linked with aesthetic sensibility, irrespective of the sensory or motor nature of the representation conveying meaning.

#### Beauty-Driven Modulation of Electrophysiological Indexes of Perceptual Learning

In a series of experiments (Sarasso et al., 2021a,b) we found that widely acknowledged electrophysiological indexes of implicit perceptual learning of sensory regularities, such as the mismatch negativity (MMN), were enhanced for subjectively more appreciated musical chords. The MMN negativity is a differential wave obtained by subtracting EEG responses to standard events to those elicited by deviant events. The MMN captures the magnitude of the update of the predictive representation of upcoming stimuli (Lieder et al., 2013). Interestingly, in the same study (Sarasso et al., 2021a) we found that trial-by-trial MMN responses to more appreciated chords were more strongly related to Bayesian Surprise, an information-theoretic index quantifying the magnitude of the update of Bayesian beliefs following each sound. This means that during aesthetic appreciation our brain is more attuned with sensory surprise and that surprising stimuli trigger greater updates of mental representation encoded in the neural hierarchy. Electrophysiological results were associated with better memorisation for more appreciated musical intervals (Sarasso et al., 2021b). As we will further explore in the following paragraph, this is likely to be mediated by the role of dopaminergic reward, signalling information gains, in learning and memory consolidation (Kang et al., 2009; Ferreri et al., 2019). It is worth noticing that the direction of the causal link between beauty perception and enhanced learning is still to be determined and not necessarily one-way (Sarasso et al., 2020a): as we will argue in the following paragraphs, the reward signal of aesthetic pleasure could be considered both a feedback and a trigger of perceptual learning.

#### Aesthetic Pleasure and Informational Value Share a Common Neurobiological Substrate

Reducing uncertainty is intrinsically rewarding (Bromberg-Martin et al., 2010; Oudeyer et al., 2016; Brydevall et al., 2018) and elicits dopamine transmission in reward related neural networks (Bromberg-Martin and Hikosaka, 2009; Brydevall et al., 2018). Indeed, informational value correlates with the activation of dopaminergic midbrain reward-related structures (Schwartenbeck et al., 2016). It was found (Bromberg-Martin and Hikosaka, 2009) that dopaminergic transmission signals the quantity of information conveyed by a task-relevant cue in a cue signalling task. The intrinsic value of information encoded by activations in reward-related areas is central to the adaptive function of curiosity which optimally redirects energy from goal-oriented behaviour to exploration and learning about the environment (Schwartenbeck et al., 2019). Interestingly, activations in the reward circuit substantially overlap with the ones that correlate with aesthetic appreciation (Blood and Zatorre, 2001; Cela-Conde et al., 2004; Kawabata and Zeki, 2004; Vartanian and Goel, 2004; Jacobsen et al., 2006). The involvement of the dopaminergic reward-related neural system in aesthetic pleasure is further demonstrated by the fact that the dopamine precursor levodopa, compared with placebo, increases the pleasure derived from music listening, while the dopamine antagonist risperidone leads to a reduction of aesthetic pleasure (Ferreri et al., 2019). Interestingly, the pleasure of music listening mediated by the activation of subcortical structures, like the Nucleus Accumbens, is significantly related to the intrinsic self-induced reward triggered by learning musical structures (Gold et al., 2019a,b). Mencke et al. (2019), as an example, explored the appreciation of atonal music and found a consistent relationship with learning mechanisms. The authors suggested that the dopaminergic activity subtending aesthetic pleasure may mediate the reward generated in response to representational models’ refinement (Mencke et al., 2019). Moreover, as indicated by studies in primates, dopaminergic activity in the reward-related network, is observed in correspondence to a certain degree of uncertainty, whereas it is lacking when the upcoming input is completely predictable (Fiorillo et al., 2003) and carries no novel information (Gold et al., 2019b). In other words, this dopaminergic-based reward mechanism may intrinsically motivate to acquire new (i.e., surprising) information (Koelsch, 2010; Ferreri et al., 2019), thus supporting the individual to tolerate the distress deriving from uncertainty and to focus on learning-oriented perception (Cheung et al., 2019; Gold et al., 2019a; Koelsch et al., 2019; Mencke et al., 2019).

#### Pain, Beauty, and the Psychopharmacology of the Endogenous Opioid System

Opioids, besides their commonly acknowledged (Machelska and Celik, 2018; Kandasamy et al., 2021) role in pharmacological analgesia (morphine is a mu-opioid agonist) and their less obvious role in attentional analgesia (self-induced mental distraction from pain; Esch et al., 2017; Oliva et al., 2021), placebo analgesia and pain empathy (Rütgen et al., 2015) have a broader and fundamental role in human sensibility and information acquisition. In general, mu-opioid activity balances pain and pleasure across sensory modalities (Meier et al., 2021).

Additionally, the brain’ crave for information (infovore behaviour) through the senses might be mediated by the opioid system (Schoeller and Perlovsky, 2016). Mu-opioid transmission signals the quantity of information conveyed by stimuli and might result in perceptual pleasure (Nadal, 2013). Indeed, beside the well-acknowledged involvement of the mu-opioid receptor system in the rewarding qualities of pleasant touch (Løseth et al., 2019), opioids might mediate perceptual pleasure across different sensory modalities (Mallik et al., 2017). Biederman and Vessel (2006) propose that inferentially rich stimuli will be preferred because they are accompanied by more activity in regions higher up in the ventral visual stream, which possess higher amounts of mu-opioid receptors. The release of endomorphins and the stimulation of mu-opioid receptors might correlate with the informational value conveyed by stimulation (Biederman and Vessel, 2006), which in turn might activate the limbic hedonic hot-spots in reward-related areas (Lacey et al., 2011; Nadal, 2013). According to these authors, the mu-opioid receptors are essential for the pleasures we derive from acquiring new information. Indeed, the limbic system might underly the experience of “liking” (Berridge and Kringelbach, 2015), which is mediated by opioids and endocannabinoid activations in the ventral globus pallidus and in the Nucleus Accumbens (Berridge et al., 2009; Berridge and Kringelbach, 2015). Indeed, this system has been suggested to be involved in aesthetic appreciation (Nadal, 2013). Evidence from psychopharmacological studies confirms this suggestion (Sarasso et al., 2020a). As an example, the opioid antagonist naltrexone dampens pupil responses to peak musical pleasure (Laeng et al., 2021). Coherently with this idea, aesthetic chills (i.e., non-thermoregulatory hedonic shivering) can be influenced by the opioid-antagonist naloxone (Goldstein, 1980), which is known to modulate stress-related amnesic mechanisms, retention and learning performances in rats (Izquierdo, 1982; Saha et al., 1991; Sajadi et al., 2007). Indeed, the motivational component of learning seems deficient in mu-opiod knockout mice (Lubbers et al., 2007). Opioid peptides are mediators of an endogenous amnesic mechanism in rats: the strength of learning is dependent upon the release of these substances (Izquierdo et al., 1980). Humans are naturally equipped with a similar endogenous analgesic and amnesic mechanism, based on opioids transmission, which mitigates the effects of the exposure to severe painful and disturbing experiences (Lanius, 2014). Importantly, the defensive mechanism of distancing from disturbing experiences may be progressively reduced during psychotherapy, with the consequence of making the patient feeling pain again. According to the Gestalt therapeutic approach, this process may have therapeutic effects and finally lead to the perception of beauty (Francesetti, 2012). Coherently, it is not surprising that the same pharmacological modifications that alter our ability to appreciate beauty can influence the perception and tolerance of pain. Crucially, opioid blockade *via* Naloxone can increase pain perception (Anderson et al., 2002), decrease pain tolerance and alter attentional analgesic mechanisms (Esch et al., 2017).

In sum, the large overlap between the biochemistry of aesthetic pleasure and perceptual learning (as well as togetherness and social support as we will discuss in Section “A Bridge Between Gestalt Field Theory and the Free-Energy Principle”) supports the link between beauty and learning, even when learning means experiencing painful or distressing feelings.

### Predictive Coding Accounts of Aesthetic Appreciation

#### We Are Embodied Models of Our World

The breakdown of the empiricist paradigm led to consider organisms (and brains) in terms of their complexity, with an emphasis on self-determination and self-organisation, as well as on the organisms’ active, open, and plastic course of evolution and growth (Guidano, 1991; Mahoney, 1991). The Free Energy Principle (FEP) and its neural implementation-Predictive Coding (PC)-are the two conceptual frameworks that consider the cognitive system in these terms, i.e., not as a passive information processor, but as an enactive (Varela et al., 2017) inferential foreseer of reality. The following paragraph will give a general introduction to the FEP and PC.

Drawing from Helmholtz’ notion of unconscious inference (Hatfield, 2002), PC describes the brain as a scientist making observations, taking in data, and generating and updating hypotheses based on that data (Hohwy, 2016). PC is derived from the free energy principle (Feldman and Friston, 2010; Friston, 2010; Clark, 2013), which posits that living self-regulatory systems must necessarily minimise variational free energy (a function of entropy) to remain alive. Analogously to Varela’s autopoietic cellular processes (Varela et al., 1974; Maturana and Varela, 1980), FEP assumes that life forms must actively construct and maintain themselves to counteract the tendency to disorder (the second law of thermodynamics) in a stochastic and entropic environment. This requires organisms to stay within a limited set of states corresponding to their phenotype and econiche. As an example, an epithelial cell will maintain a given form and dimension, just as a seagull will spend most of his time out of the water. Contrarily, a fish will stay mostly underwater, for the simple reason that a fish that spends most of his time out of the water is a dead fish. In this sense an agent is a model of its world. Minimising free energy (formally, the upper bound on surprise) means to minimise surprising sensory states, e.g., in case of a fish, to stay out of water. Another way of saying this is that life forms must minimise the uncertainty related to the states they occupy. Again, it is not adaptive for a fish to be uncertain regarding the probabilities of being in or out of water. This autopoietic (Friston, 2013) process of seeking a limited number of “attractor” states is called (local) ergodicity (Ramstead et al., 2018). The main insight of the free energy principle is that ergodicity is an intrinsic property of self-regulating biological systems that emerges through modelling the world (Friston, 2010; Hirsh et al., 2012). Gestalt therapy similarly hypothesises that self-regulation leads to a limited set of modes and that novelty is sometimes resisted: “the resistance protects him [the patient] by ensuring that his habitual mode of self-regulation remains intact” (Yontef and Jacobs, 2005; p. 311).

The simplest self-regulatory mechanism in stationary organisms such as plants corresponds to homeostasis, which keeps the internal milieu constant. Movement, however, brings in the need for modelling future states (Van de Cruys and Wagemans, 2011a). Animals move and act in the environment to eat and find shelter. Homeostasis thus requires non-stationary life forms to predict future states and upcoming stimuli. Therefore animals, especially those on the higher end of the evolutionary scale, whose predictive capabilities are greatly enhanced, constantly make predictions by inferring the causes of sensations. These predictions, or predictive representations, constitute a hierarchical generative model of the hidden causes of the sensations (i.e., states of the world; Friston, 2003). Similarly to Gestalt therapy, PC entails a boundary between the mind and the “real world,” which in principle cannot be directly accessed but only guessed. The concept of a “Markov blanket” (comprising action and sensory states) provides a formal basis for this boundary that separates internal (mental) representation and external hidden states (Hohwy, 2016). External states are hidden from internal states in the sense that they can only be seen indirectly by virtue of their causal dependencies. Biological systems are ergodic dynamical systems that possess a Markov blanket (Gallagher and Allen, 2018). The function of a Markov blanket is to minimise the difference between the generative model of the world (i.e., the predictive representation of the causes of sensory inputs) and incoming sensory data (Friston, 2013). To simplify, we could say that we constantly make and update a predictive representation of our environment (Friston and Kiebel, 2009), our bodies and future actions (Limanowski et al., 2018). Conscious perception, and even our sense of being a self (Limanowski and Blankenburg, 2013; Limanowski and Friston, 2018), is the best explanation (or best prediction) we can come up with of sensory data (Havlík et al., 2017). In this sense, we and the world that surrounds us are nothing but inferences constantly drawn from somatosensory embodied processes (Limanowski and Blankenburg, 2013).

The long-term imperative of minimising surprising sensory states is thereby achieved in the short term *via* the minimisation of prediction errors (Friston, 2010), i.e., the mismatches between actual and predicted states. Both action and perception aim at minimising prediction errors, either by changing the world or by changing our internal representation of it, respectively. Perception “explains away” prediction errors by adjusting predictions according to unpredicted sensations. This is called predictive coding (PC). Along the neural hierarchy, “conditional expectations of perceptual causes” or predictions (priors) are generated and transmitted from higher associative areas to lower levels, where they are compared with incoming inputs. These “top-down” predictions inhibit “bottom-up” sensory inputs that are coherent with them, leaving only the unpredicted mismatches between predicted and incoming data (i.e., prediction errors) to propagate from lower areas upward along the sensory hierarchy. PC shares many intuitions with Gestalt therapy. As an example, in their foundational book on Gestalt therapy published in 1951, Perls, Hefferline, and Goodman reported the following description of the intentionality of contacting, which might sound similar to what we just described here: “Now what is selected and assimilated is always novel. The organism persists assimilating the novel, by change and growth […] what is “unlike” that becomes “like”; and in the process of assimilation the organism is in turn changed. Primarily, contact is the awareness of, and behaviour toward the assimilable novelty; and the rejection of the unassimilable novelty. What is pervasive, always the same or indifferent is not an object of contact” (Perls et al., 1951; p. 230).

Alternatively, action reduces prediction errors by actively ensuring that predictions are fulfilled. When we act we sample sensory inputs so that they conform to predictions (i.e., active inference). According to this principle the action-perception cycle is a matter of uncertainty reduction (Sarasso et al., 2020a), or as Humberto Maturana claimed: life should be understood as a process of knowing (Ruiz, 1996).

A crucial question then emerges, which might be of interest also for the study of change processes in psychotherapy. In the words of Karl Friston: “If proprioceptive prediction errors can be resolved by classical reflexes or changing (proprioceptive) expectations, how does the brain adjudicate between these two options?” We previously described aesthetic appreciation as a feedback signalling the transient (informational) profitability of directing neural resources to changing predictive representations rather than acting based on previous routines (Sarasso et al., 2020a, 2021c). For this reason, as we will discuss in the following paragraphs, the perception of beauty might be intimately linked with therapeutic change.

#### Affect and Predictions: “I Feel Good, I Knew That I Would, Now”

Agents in a volatile environment are not only equipped with predictions but also with second-order predictions regarding the accumulated evidence in favour of their predictions. This “how am I doing” might be intimately linked with affect (Hesp et al., 2021). Emotions and moods provide feedback of our coping efficacy in the interaction with the environment (Frijda, 1986; Reisenzein, 2009; Clark et al., 2018) and are hypothesised to have the adaptive role of adjusting learning rates according to environmental changes (Joffily and Coricelli, 2013). Rates of mismatch reduction relative to behavioural goals (i.e., the distance between a desired state such as drinking water, and the current state) have long been proposed to be at the root of emotional valence, the positive-negative dimension of emotions (Carver and Scheier, 1990). However, since goals are only one important type of prior expectations (desired states are just one among many types of expected states) we can generalise this view and hypothesise that affects such as pleasure and distress reflect the dynamics in prediction error reduction (Joffily and Coricelli, 2013). According to this view, emotional valence relates to confidence in one’s own internal model of the world (Bodenhausen et al., 1994; Gasper and Clore, 2002; Seth and Friston, 2016; Badcock et al., 2017; Clark et al., 2018). In computational terms, emotional valence corresponds to the rate of change of free-energy over time (proper emotions such as fear or hope can only be explained once the second time-derivative of free-energy is taken into account; Joffily and Coricelli, 2013).

Unpleasant emotions arise when free energy (uncertainty) increases over time. On the contrary, when PE reduce over time, we experience a positive affect (Van de Cruys, 2017): we feel good when the first time-derivative of free energy is negative (Joffily and Coricelli, 2013). “We are happy when we are growing” as the Irish poet Yeats wrote, which, in Gestaltic terms, means that we are happy when we are assimilating and integrating novelty at the contact boundary between the organism and the environment. As it has been already suggested above, and as will be further discussed in the next paragraph, aesthetic pleasure might have a fundamental epistemic role in uncertainty reduction dynamics. What we are suggesting here is that, in a sense, all kinds of pleasures have an epistemic status since they might be related to prediction error reduction.

#### Beauty as a Meta-Learning Feedback From Prediction Error Dynamics

As many enactivists (Noë, 2005) suggest, perception does not serve only for recognition and identification, but “is also reward-oriented, hedonic, aesthetic, and affective in the broadest sense—and in ways that suggest that we may enjoy (and seek) perceptual surprise” (Gallagher and Allen, 2018). Biological and artificial intelligent systems must develop an intrinsic feedback on learning gains in order to recognise stimuli which maximise epistemic value, to direct attention to informative and “learnable” stimuli and to modulate the active sampling of sensory input (Gottlieb et al., 2013). For this purpose, the brain generates intrinsic reward to learnable and novel stimuli with high informational content (Oudeyer et al., 2007). Experiences are indeed more pleasurable when they can be assimilated while still providing novel information to the observer (Biederman and Vessel, 2006). This hypothesis has been recently demonstrated by Grzywacz and Aleem (2022) who have shown that the absolute quantity of information computed as “Fisher information” (a measure of uncertainty-reducing information quantifying “how much can be learned” from a sensory stimulus) can modulate aesthetic preferences for certain sensory patterns.

In terms of FEP the information conveyed by stimuli is quantified by the magnitude in the update of beliefs brought by prediction errors, which can be computed as the information-theoretic index named Bayesian surprise (Baldi and Itti, 2010). As it has been suggested before, we hypothesise that beauty is how we experience the update of beliefs which “explains away” prediction errors (Sarasso et al., 2020a). Since Gestalts can be thought as specific predictions (prior beliefs in Bayesian terms) used by the visual system to efficiently disambiguate visual input (Costa and Wagemans, 2021), we could alternatively say that we find something beautiful when a new Gestalt brings order into chaos. Therefore, the “achievement of a strong Gestalt” (Perls et al., 1951, p. 232) guided by aesthetic criteria at the root of the therapeutic intervention corresponds to a large update in prior beliefs which we are naturally equipped to sense as beautiful (Sarasso et al., 2020a). Aesthetic pleasure, however, can only follow an effortful tolerance of defied predictability (Van de Cruys and Wagemans, 2011b): “The effort of mental work one has to do to cope with the prediction error is a condition *sine qua non-for* receiving perceptual pleasure of a Gestalt formation (prediction error reduction)” (Kesner, 2014; p. 6). This resonates with the idea that aesthetic experiences are both disruptive and transformative at the same time (Pelowski and Akiba, 2011; Kesner, 2014).

Similar ideas have been put forward by numerous authors. Van de Cruys et al. (2021) further emphasise that meta-predictions on the expected learning rate should also be taken into account: both the gradient and the unexpectedness of prediction error reduction are crucial for experiencing pleasurable aesthetic emotions. Intense aesthetic appreciations mark unexpected increases in error reduction rates (Van de Cruys and Wagemans, 2011b; Van de Cruys, 2017). Chetverikov and Kristjánsson (2016), differently propose that it is not necessary to consider meta-predictions in the genesis of positive aesthetic emotions in response to prediction error reduction. According to these authors, the update of perceptual predictions yields hedonic feedback that is inversely weighted with prior probabilities (the probability assigned to a given prediction before stimulation and the consequent update in predictions) of these newly acquired predictions, so that in highly predictable environments correct predictions will trigger only mild positive affect, while new predictions in unpredictable environments are marked with a strong positive affect. What matters is not prediction error reduction *per se*, but learning, since the magnitude of the update of predictions (distance between priors before and after stimulation) depends on its prior probability.

The proposed link between predictive processing dynamics, knowledge acquisition, and the experience of beauty raised several concerns among those who consider aesthetic experiences mainly as an emotional rather than a cognitive/epistemic experience (Armstrong and Detweiler-Bedell, 2008; Brielmann et al., 2021). It is worth to acknowledge that the hypothesis linking prediction error reduction and aesthetic appreciation is susceptible to be misinterpreted as purely cognitive, or “un-empathic” (Sarasso et al., 2020a). As we discussed in Sarasso et al. (2020a), this interpretation is incorrect, as prediction errors do not necessarily fall within what is usually considered as the cognitive domain. Indeed, predictive coding defies the classic distinction between cognitive/conceptual and affective domains (Barrett, 2017). Affects result from one special kind of interoceptive inference which leads to predictions and prediction errors as any other perceptual or cognitive act (Seth, 2013). Similarly, embodied sensory-motor resonances are conveyed by prediction errors in mirror areas and can result in the update of predictions just as any other sensory information (Kilner et al., 2007). The update of predictions can therefore correlate with feelings at a phenomenological level and do not necessarily trigger changes along more abstract and verbal hierarchies of the generative model. Along these lines, if we conceive knowledge as the result of an embodied, enactive and emotional experience (Immordino-Yang and Damasio, 2007; Fuchs and Koch, 2014), the possible link between learning and beauty perception becomes less “cognitive” and more “empathic” (Gallese and Sinigaglia, 2011; Stamatopoulou, 2017).

#### I Know I Don’t Know: Paradoxical Strive for Uncertainty

Nietzsche first evidenced the ambiguity and paradoxical nature of knowledge acquisition for “the seeker of knowledge forces his spirit to recognise things against the inclination of the spirit” (Nietzsche, 2010; p. 259). For Nietzsche “knowledge appears as renunciation of the happiness of a sturdy and vigorous illusion” (Nietzsche, 1982; p. 184). FEP posits that the brain abhors informational surprise and that it minimises it by (A) acting, enhancing the statistical likelihood of sensory samples, or else (B) by revising inferences in the light of experience and updating “priors” to reality-aligned “posteriors”; (C) optimising the complexity of our generative models of the causes of ambiguous sensations. Although, as explained above, our brain is in the game of maximising the evidence of its internal model of the world while minimising the uncertainty associated to sensory states (i.e., prediction errors), organisms and especially humans do not avoid all uncertainty. In principle, prediction error minimisation, by generating action-perception cycles that minimise surprising interactions with the world, would lead us to seek out for stimulus-free dark rooms (the so-called dark room paradox; Friston et al., 2012b). Lively organisms, however, do not actually look for complete absence of prediction errors by taking refuge in dark rooms, for the simple reason that dark rooms would result into highly unpredictable states, since humans developed both phylogenetically and ontogenetically in an econiche that is very different from a dark room (Sun and Firestone, 2020). On the contrary, experience teaches us to expect to encounter and “explain away” prediction errors (Chetverikov and Kristjánsson, 2016; Van de Cruys, 2017). McReynolds (1971) argued that humans maintain an expected rate of cognitive experience (the process of assimilation of new percepts into mental models) through exploration. The two drives of such intrinsic cognitive motivation have been identified by McReynolds as the minimisation of “unassimilated perceptual material” and the optimisation of innovation rate (update of mental models). In mathematical terms, since birth we build second-order expectations over the (positive) rate of prediction error reduction. We track and learn to expect certain non-zero rates of change in prediction errors, which makes us inclined to explore and learn (Van de Cruys, 2017).

The paradoxical search for learning gains is adaptive (Oudeyer et al., 2016): “The paradox is that expecting uncertainty and inviting chaos (what we could call *radical curiosity*) can lead one to perceive new layers of regularities in reality” (Van de Cruys et al., 2021, p. 31). We need to encounter some prediction errors to get better at predicting our environment. In other words, to survive as individuals and as a specie, we need to expect to keep learning something new. In this sense, we are oriented to growth, as Gestalt therapy posits. We instinctively know that we could better explain reality and we foresee that we don’t see yet. We know there is a model, which could provide us better explanation for what we observe, we just don’t know the model yet. Mark Miller has wonderfully expressed this point in his discussion on metastable dynamics (Miller et al., 2021; p. 9): “agents also tend to actively destroy fixed point attractors therefore inducing instabilities and creating peripatetic or itinerant (wandering) dynamics (Friston et al., 2012a).” Predictive organisms do not only seek to maximise error reduction but are also driven to reduce error at a particular rate (Kiverstein et al., 2019). They are willing to disrupt their own fixed-point attractors (habitual policies) to explore just-uncertain-enough environments that are ripe for long term prediction error minimisation (Kiverstein et al., 2019; Seth et al., 2020; Van de Cruys et al., 2020). Organisms “that live in complex dynamic environments will benefit from remaining at the edge of criticality between order and disorder, between what is well-known (and reliable) and the unknown (and potentially more optimal) […] Metastability is the consequence of two competing tendencies of the parts of a system to separate and express their intrinsic dynamics and to integrate and coordinate to create new dynamics” (Miller et al., 2021; p. 9). The authors go on explaining that “Metastability is intrinsically linked to affective value.” Indeed, when a particular econiche ceases to yield negative error slopes, negative affects signals to the organisms that they need to destroy their own fixed-point attractors to favour exploration. On the contrary, when errors accumulate over time because of unmanageable complexity, negative affects prompt the agent back to routinary behaviour that is already well-known and highly reliable (Miller et al., 2021).

Our thirst for surprise and change (i.e., update of beliefs to minimise prediction errors) might be at play also in pathological conditions, in such a way that even psychopathology preserves an intentionality for contacting (see Appendix A). However, the tension toward growth, novelty, and explorations seems sometimes to fade away. As an example, it has been suggested that some pathologies, such as Major Depression (MD), are “better safe than sorry” adaptive responses to adverse social events that minimise the likelihood of the occurrence of surprising interpersonal interactions (Badcock et al., 2017). This behaviour might be caused by the fact that, following ineffectual attempts to alleviate interpersonal difficulties (e.g., social uncertainty; loss) in competitive or adverse social contexts (Badcock et al., 2017), the patient learned that it is difficult to reduce uncertainty (i.e., prediction errors) through goal-directed behaviour, therefore inhibiting the expectation to reduce prediction errors through exploration and exploitation behaviours (Constant et al., 2021). The organism learns that action cannot reduce social distance in its social context. Therefore prediction errors, in this case the difference between desired and actual (adverse) social outcomes, remain high and continuously affect the organism. Depressed patients are strongly convinced that their environment is very uncertain (Smith et al., 2021). For this reason, they learn to be helpless (Abramson et al., 1978) and reduce energy expenditure (Barrett et al., 2016; Kiverstein et al., 2020) through “sickness behaviour” (Stephan et al., 2016; Badcock et al., 2017; Quadt et al., 2018). In such cases, recovery in psychotherapy might be triggered by “relearning” through the therapeutic relationship a positive expected rate of error reduction through experience and engagement with the world (Miller et al., 2021). Depression itself might be a desperate adaptive attempt to reduce prediction errors (i.e., social distance) with increased sensitivity to social signals and signalling behaviours that either garner support (e.g., reassurance seeking) or defuse conflict (e.g., submissive behaviours; Badcock et al., 2017). In this sense the intentionality of contacting might be preserved even in severe depression.

In short, a “healthy” predictive agent seeks rather than avoids novelty (Van de Cruys, 2017). In this learning process, curiosity has a fundamental adaptive value, since it allows us to explain ever deeper layers of regularities in the environment. Gestalt therapists refer to this strive for uncertainty as “cultivated uncertainty” or “willingness to be uncertain” and consider this therapeutic attitude a fundamental, if not necessary, ingredient for therapeutic change (Staemmler, 1997, 2006). For this reason, we believe that the investigation of the different motivational components that facilitate the openness to uncertainty is important for the evolution of psychotherapy (Francesetti, 2019b). Along this line, we should try answering the following question: what is the role of affect, in particular of aesthetic pleasure, in teaching us to expect uncertainty reduction?

#### Beauty Makes Us Curious (and Less Anxious)

Not all uncertainty results in learning and change, since social and motivational support is needed to overcome the tendency toward a limited habitual set of sensory states. Aesthetic pleasure might represent a peculiar case of motivational support sustaining the integration of novel states in the predictive models of the self-evironment. A state characterised by an increase in prediction errors, corresponding to sensory uncertainty and signalling the need to update sensory or motor predictions, will transitorily produce negative emotions and arousing sensations (Joffily and Coricelli, 2013; Braem et al., 2015). Humans are typically uncertainty averse (Carleton, 2016) and are willing to pay to reduce uncertainty (Lovallo and Kahneman, 2000). This could be the case of therapy where unformulated and unspeakable chaotic proto-feelings urge us to seek help to become feelings (Francesetti and Roubal, 2020). Arousal (norepinephrinergic neuronal excitations; Barrett, 2017) is what in Gestalt therapy is referred to as excitement (see Glossary). Arousing signals within the amygdala, other limbic regions, and the cerebellum are forwarded to the cortex to correct the generative model of sensory inputs (Buckner et al., 2011; Haber and Behrens, 2014; Barrett, 2017). Arousing error signals associated with increases in amygdala activations (Whalen, 1998; Wilson-Mendenhall et al., 2013) can thus be considered a learning signal (Li and McNally, 2014), but do not necessarily lead to learning. When adequate support is lacking in the organism-environment field, excitement/arousal can result in anxiety (Perls et al., 1951): “excitement that should lead to the contact becomes undefined energy” (Spagnuolo-Lobb, 2015; p. 8). Interestingly, it has been suggested that clinical and subclinical anxiety is related to the intolerance of emotionally arousing uncertainty (Dugas, 1997; Bishop, 2007; Carleton, 2016; Anderson et al., 2019) brought by prediction errors (Del Popolo Cristaldi et al., 2021). Intolerance of uncertainty, both at a subjective and neural level, in turn was shown to be detrimental for learning (Hein et al., 2021).

Curiosity, on the contrary makes uncertainty and arousal not aversive when accompanied by an appraisal of coping potential, that is one’s expectation regarding the ability to understand or deal with prediction errors, in the sense of making it predictable or meaningful again (Silvia, 2005). Graf and Landwehr’s (2015) hypothesised that stimulus processing needs a sufficient processing motivation triggered by a perceiver’s need for cognitive enrichment or the stimulus’ processing affordance. Only when this motivation is high the engagement of elaborate perception resulting in aesthetic interest is possible. Neurocomputational research, and neuroaesthetics particularly, has renewed the interest on the intrinsic motivational aspects of curiosity. Schmidhuber (2009), for example defines it as if follows: “Curiosity is the desire to create or discover more non-random, non-arbitrary, truly novel, regular data that allows for compression progress because its regularity was not yet known. This drive maximises “interestingness,” the first derivative of subjective beauty or compressibility, that is, the steepness of the learning curve. It motivates exploring infants, pure mathematicians, composers, artists, dancers, comedians, yourself, and recent artificial systems” (p. 1). Curiosity is a sort of heuristic for maximising learning progresses (Oudeyer et al., 2016). Not surprisingly, indeed, curiosity is the primary promoter of learning and change (Kang et al., 2009).

Here we propose that, in a clinical context, the aesthetic attentional attitude triggered by the expectation of aesthetic reward (i.e., finding beauty in the therapeutic encounter, or, else said, finding it beautiful) can facilitate the attainment of the psychological distance necessary to fully embrace arousing and potentially anxiogenic experiences (Menninghaus et al., 2017). How might aesthetic sensibility help us to shift from anxiety to curiosity when facing arousing unpredicted stimuli will be discussed in the following paragraph.

One of the first influential views on curiosity and aesthetics is Berlyne’s optimal level account (Berlyne, 1970), arguing that organisms seek out stimuli with medium level complexity or novelty, to keep their arousal at an optimal, pleasing level. More recently, along these lines, it has been suggested that aesthetic pleasure support natural curiosity (Schoeller, 2015) and the drive for knowledge acquisition and meaning (Perlovsky, 2006). Within the PC framework, aesthetic emotions might motivate the paradoxical drive for uncertainty since this type of self-generated reward teaches us to expect steeper prediction error reduction slopes (Sarasso et al., 2020a).

The effect of expecting beauty is openness to experience. Contemplation of the beauty is often compared to the concept of *absorption* proposed by Tellegen and Atkinson (1974), which is described as openness to experience in which attentional amplification engage the totality of available mental (perceptive and representational) and executive (motor) resources of the individual.

An aesthetic attitude allows the organism to tolerate a momentary state of uncertainty for the seeking of new knowledge instead of reacting according to previously stored knowledge. In Sarasso et al. (2020a) we discuss evidence demonstrating that aesthetic pleasure emerges in correspondence with an inhibition of motor behaviour (i.e., minimising action), promoting a simultaneous attentional perceptual enhancement, mediated by synaptic gain modulations at the level of sensory cortices (i.e., optimising learning). Accordingly, we suggest that the perception of beauty may represent an hedonic feedback over learning progresses, motivating the individual to inhibit previously acquired motor routines to seek novel knowledge acquisition. Beauty perception might represent the motivational drive that pushes us toward novelty. This motivation is intrinsic to the process of new Gestalts formation (beliefs update in PC terms): “the anxiety is tolerated […] because the disturbing energy flows into the new figure” (Perls et al., 1951; p. 233). The founders of Gestalt therapy then go on writing that the ability to tolerate uncertainty-driven anxiety “comes from previous experience having been assimilated and growth achieved.” Beauty is a self-generated reward elicited by the assimilation of new experiences that teaches us to expect learning progresses, i.e., to get better and better at explaining what we observe. In PC terms it allows us to form a second-order prediction (hyperprior or meta-prediction) on learning progresses. Beauty might make us naturally curios about the hidden causes of the world (Perlovsky et al., 2010; Mirza et al., 2018). Without aesthetic rewards, we would avoid uncertainty and novelty, act to escape the anxiety associated to novelty and become emotionally and aesthetically numb with respect to others and the environment (Hillman, 1988). This might be the reason why aesthetic emotions are important in psychotherapy. As we will discuss in detail in Section “Distancing to Embrace,” curiosity, and, indeed, also an aesthetic attitude [see Menninghaus’s distancing-embracing model in Menninghaus et al. (2017)], help the clinician to keep some distance from the impulse to act triggered by the proto-feelings circulating in the therapeutic field (Francesetti and Roubal, 2020).

## A Bridge Between Gestalt Field Theory and the Free-Energy Principle

In the following section we will try to bridge the gap between the notion of aesthetic pleasure presented in Section “Aesthetics and Knowledge/Change” and Gestalt therapy intuitions. Our aim is to suggest a plausible neurophysiological correlate of therapeutic change mediated by the therapist’s aesthetic attitude. In Section “Suffering of Experience: Gestalt-Phenomenological Approach to Psychopathological Fields,” we described how conservative field forces are perceived as sensations, impressions or atmospheres in the form of unprocessed proto-feelings in rigid psychopathological fields (Francesetti and Roubal, 2020). We believe that modern neurocomputational modelling in cognitive sciences can account for unprocessed feelings and the reason they remain inaccessible and anesthetised in psychopathological fields. Proto-feelings could be defined as prediction errors that still need to be “explained away” by the update of the generative model of the causes of sensory states [no matter whose model, the therapist’s or the patient’s, for at a first stage of perception they are still undifferentiated (Damasio, 2012) in the experiential field (Section “The Field Perspective: A Dive Into the Undifferentiated”)]. Within this framework, based on the FEP described in Section “Aesthetics and Knowledge/Change,” unprocessed proto-feelings and sensations transiently arise the uncertainty associated to sensory inputs and urge the organisms, or the system of two or more organisms, to reduce it, either by action or learning (i.e., updating the generative model). This corresponds to free energy minimisation in the FEP, which substantially maps onto what Gestalt therapists call the intentionality of contacting. Changes in the shared generative model can restructure the experiential field organisation and lead to therapeutic change. Coherently, some authors defined therapy as the generation of new predictive representations: “Moments of creative not-knowing may emerge and hence the need for active exploration, innovation and generative possibilities… [therapy] may encompass both strategies to engage predictive processing neurodynamics—sampling new sensory input through action (active inference) and shaping the internal models of the world (prediction signals) through meaning-making” (Vaisvaser, 2021; p. 5). New predictions are therapeutic when they can explain a greater deal of prediction errors which can be successfully integrated into experience or otherwise remain unexplained when sufficient interpersonal support lacks. People might find relief in this process since new priors can explain a greater deal of prediction errors, thereby reducing the overall uncertainty and intersubjective chaos they are exposed to. Indeed, as we discussed in Section “Affect and Predictions: “I Feel Good, I Knew That I Would, Now”,” we are equipped with an hedonic feedback signalling uncertainty reduction dynamics: feeling good might be intrinsically linked to the reduction of prediction errors over time and at a certain rate (Joffily and Coricelli, 2013).

Change and the update of predictive representations, however, follow a necessary momentary rise in sensory uncertainty, which could be operationalised as a sensory upweighting of affective and sensory prediction errors (i.e., proto-feelings) mediated by attentional dynamics *via* cortical gain control (Feldman and Friston, 2010; Clark, 2013). Such neuromodulatory gain control might correspond to a modulation of the excitability of neuronal populations encoding prediction errors (Feldman and Friston, 2010; Shipp et al., 2013). Sensations, impressions and atmospheres must first be felt to further be processed, shared and communicated. Therapeutic change is not only a cognitive act, since it is motivated by affective value and it implies the embodied attunement to the sensory phenomena emerging in each therapeutic situation (Fonagy and Target, 1997; Holmes and Nolte, 2019). The delay between the moment unprocessed sensory impressions are up-weighted and the moment this triggers an update in the predictive model can be distressing, since the model of “what is happening to me” cannot account for something that is clearly happening to me. The reason this process is both arousing and distressing lies in the inverse relation between affective value and uncertainty described in Section “Affect and predictions: “I feel good, I knew that I would, now”.” To provide support in tolerating such distress might be the main function of clinical theories, settings and practices. It is well-acknowledged that even at a neurophysiological level, sensory inputs (i.e., prediction errors) that are too chaotic and unlearnable, with a lower estimated signal to noise ratio are attentionally down-weighted (Auksztulewicz and Friston, 2015; Ronga et al., 2018; Sarasso et al., 2020b). The ability to resist this tendency by shifting back attention to confused impression, sensations, and chaotic feelings might be a key therapeutic ability. Field therapy changes the focus from the patient to the therapist. It is the therapist’s experience of the field conservative forces that changes in the therapeutic process. During psychotherapy “[…] the therapist is continuously dealing with the uncertainty of the unfolding field. He/she needs to be able to tolerate not knowing and to be ready to change direction according to the field’s forces” (Francesetti and Roubal, 2020; p. 10). In simple terms, feeling proto-feelings that previously could not be felt, can trigger therapeutic change. According to a synergetic scheme (Ciompi and Tschacher, 2021), as we will discuss below, this introduces new inputs into the complex dynamics of self-organisation, which will eventually result in new attractor states “enslaving” self-organisation along new patterns. A transient rise in sensory uncertainty (i.e., Free energy) might require the metastable patient-therapist system to move toward new attractor states.

Processing proto-feelings, however, needs social, relational, experiential, and attentional support. Most importantly, in our view, aesthetic reward might provide additional motivational support to tolerate uncertainty. Moreover, we need others to “turn up the volume” of sensations that could not be assimilated and integrated in our model of what is causing them. In the following Section “Two Bodies Are Better Than One,” we discuss how solitude and togetherness influence attentional dynamics and the neurophysiology of perceptual learning and change.

In the following paragraphs we will try to merge the theoretical constructs of Gestalt therapy, field theory and predictive coding to discuss how the aesthetic sensibility of the therapist might be among the factors supporting the transformative intentionality of contact of the field.

### Two Bodies Are Better Than One

As we discussed in Section “Psychopathology: Growing With Unprocessed Experiences,” disturbing and dissociated proto-feelings that remain unformulated in psychopathological fields need another body to be sensed with full aesthetic sensibility, and, secondly, to be consciously experienced, communicated and signified, thereby reorganising the field of experience. Gestalt therapy posits that we need someone else next to us to experience what could not be experienced (Francesetti, 2019a). Else said, updating the generative model of what is happening to us necessitates the presence of others. New Experiences (i.e., new Gestalts) need togetherness according to this phenomenological account of therapeutic change. Is there any neurophysiological evidence for the role of togetherness in allowing the experience of novel sensory information? Evidence from the Shared attention effect and opioid transmission might provide us a tentative answer.

Sharing attentional targets with others (Koike et al., 2016) favours the encoding of novel information, emotions, and sensations (Decety and Fotopoulou, 2015). It has been demonstrated that sensory outcomes of shared experiences are amplified (Boothby et al., 2014; Shteynberg et al., 2016), and that people devote greater cognitive resources to co-attended stimuli (Shteynberg and Apfelbaum, 2013; Shteynberg et al., 2014). Consequently, shared experiences undergo deeper perceptual processing (Craik and Lockhart, 1972; Craik and Tulving, 1975). Shteynberg and colleagues demonstrated that sharing experiences with others can foster emotional intensification (Shteynberg et al., 2014) and memory, e.g., the subsequent recall of a list of co-attended words (Shteynberg, 2010). Sensory information that are shared with significant others, such as in-group members (Shteynberg, 2010; He et al., 2011; Eskenazi et al., 2013), familiar relationship partners (Boothby et al., 2017), and caregivers (Reid et al., 2004; Tomasello et al., 2005) are particularly likely to attract attentional resources. Indeed, “psychological closeness” (i.e., strangers vs. friends; Boothby et al., 2017), just like physical distance, between co-experiencers is among the major factors that modulates the amplification of shared experiences (Boothby et al., 2014).

Altogether, these findings are referred to as *shared attention effects* (Shteynberg, 2015). We previously suggested (Sarasso et al., 2022) that the intensification of shared experience may be interpreted as the result of a sensory up-weighting driven by co-presence and implemented through the disinhibition of the post-synaptic gain of pyramidal cells encoding prediction errors (Auksztulewicz and Friston, 2015; Heilbron and Chait, 2017).

Furthermore, as we discussed above, the μ-opioid receptor (MOR) system is known to interact with the dopamine system in brain regions implicated in reward processing (Hagelberg et al., 2002; Lintas et al., 2011). Namely, activations in the limbic hedonic hotspots triggered by opioid transmission underly aesthetic pleasure and the “liking” experience more in general. The shared biochemical substrate between (aesthetic) pleasure and learning/amnesic systems (Section “Pain, Beauty, and the Psychopharmacology of the Endogenous Opioid System”) is a piece of evidence suggesting the importance of aesthetic sensibility in allowing learning and change in a psychotherapeutic setting vs. the maintenance of a certain rigid experiential field. Furthermore, the opioid mediated learning system might not be independent from social support and the presence of others. Loneliness and separation might impair learning mediated by the endogenous opioid system. E.g., it has been evidenced that separation and loss (i.e., parental death, parental separation, or divorce) affect the functionality of the endogenous opioid system, and the deficit of the opioid system may explain separation anxiety, respiratory anomalies and panic disorder (Preter et al., 2011; Preter and Klein, 2014). Indeed, just as learning and pleasure, social attachments may reflect an opioid mediated addictive process in the brain (Panksepp, 1998). As suggested by Katz (2005), opioid transmission subserves the felt hedonic core of mammalian prosociality and of consummatory pleasure more in general. The MOR system is proposed to interact with oxytocin and dopamine in social bonding and social reward processing (Depue and Morrone-Strupinsky, 2005; Tops et al., 2014). E.g., a 15 min separation from the dam during postnatal days can induce long-term changes in brain opioid and opioid receptor densities in rats (Ploj et al., 2003). On the contrary, engaging in affiliative interactions such as social play and social grooming, is associated with endogenous μ-opioid release in the brain reward circuitry in both rodents and primates (Panksepp and Bishop, 1981; Keverne et al., 1989; Vanderschuren et al., 1995). The effect of prosociality on the disposability of endogenous opioids, given the involvement of the MOR system in motivating the processing of novel information, might be interpreted as the neurochemical substrate of the influence of social support on perceptual processing dynamics.

In sum, the upweighting of sensory information resulting in learning and change, aesthetic pleasure and social bonding might be concurrent and mutually interacting factors influencing the possibility of sensing novel experiences. We speculate that the hypothesised link between learning, beauty and sharing might be subserved by common physiological substrates.

### Distancing to Embrace

Besides social support, other motivational variables might influence our ability to tolerate uncertain proto-feelings for the seek of long-term prediction error minimisation.

**FIGURE 1:**
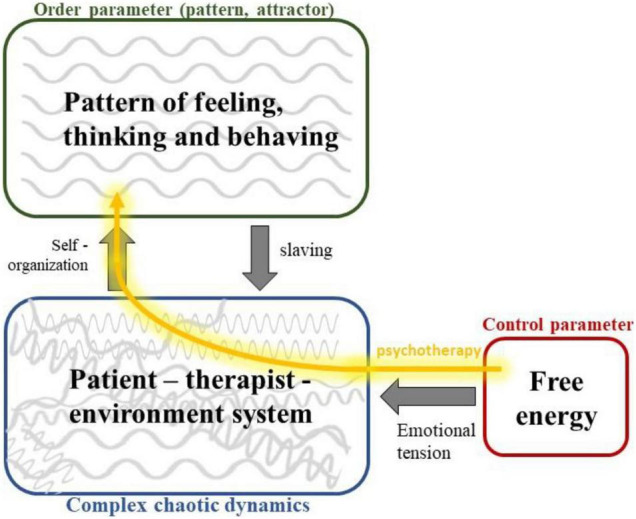
Synergetics applied to psychological change. When Free energy (i.e., uncertainty/prediction errors) reaches a critical level, the usual pattern of feeling, thinking, and behaving can no longer coordinate and synchronise system dynamics. These patterns, which in FEP terms correspond to a shared generative model (see Section “Merging Predictions in the Pre-subjective Chaos”), can be disturbed and altered by critically increasing energetic tensions, so that the organism or system of organisms self-organises into a novel metastable order parameter. Change occurs (either in the direction of healthy or psychopathological development) at this point of bifurcation, when the update of the order parameters prevails over the slaving of the complex dynamics. This non-linear shift is promoted by psychotherapy, which renders it possible to introduce into the system uncertainty, novelty and the necessary distance to assimilate surprise without reproducing and re-enacting previously acquired behavioural patterns.

A transient rise in affective and sensory prediction errors can be disturbing and sometimes unbearable for subjects involved in an experiential field such as the patient and therapist. Surprising prediction errors raise the level of anxiety and favour rigid experiential and relational patterns, which could be seen as a “fast track” or automatic way to level prediction errors back to homeostatic levels (i.e., attractor states). From the point of view of the therapist: “Intervention at this point is usually a way of avoiding the anxiety related to what is emerging. So, I try not to take any action towards the client based on what comes first, on the first wave of my experience. That way, I am introducing a higher degree of freedom into the field. Acting according to the first feeling would probably support the repetitive patterns, since it is the way along which I am taken by the absence that characterises the psychopathology of the field. To act now would carry a high risk of making the enduring relational themes (Jacobs, 2017) circulate once more, and a high risk of re-traumatising the client” (Francesetti and Roubal, 2020; p. 125). In this case, the usual pattern, or “order parameter,” “enslaves” the complex dynamics of the system by avoiding surprising sensory states (see Figure 1). However, when the transient rise in prediction errors (i.e., uncertainty/free energy/surprise) surpasses a critical level, the order parameter cannot synchronise and coordinate the complex system dynamics and elements anymore. Growing uncertainty levels, which we subjectively feel as a growing emotional tension (Tschacher et al., 2017), can push the therapist/patient system toward novel and more (meta)stable functional patterns of feeling, thinking, and behaving (Tschacher and Haken, 2007; Ciompi and Tschacher, 2021). Free energy might act as a control parameter at the point of bifurcation where the system “chooses” between rigid or novel attractor states (Ciompi and Tschacher, 2021). Thereby, a transient rise in uncertainty needs to be accepted and assimilated for change to occur.

Biological and artificial intelligences need a feedback mechanism that pushes them to actively seek informationally profitable surprising sensory states (Kaplan and Oudeyer, 2004; Oudeyer et al., 2007; Gottlieb et al., 2013). Therefore, similarly to any costly, effortful, and risky exploratory behaviour, also attending and tolerating sensory uncertainty is rewarded by an intrinsically generated hedonic feedback (FitzGibbon et al., 2020). Moreover, as any reward, the expectation of self-generated aesthetic pleasure might motivate the paradoxical search for sensory uncertainty (Sarasso et al., 2020a, 2021a,c). We suggest that aesthetic pleasure allows toleration of attentionally up-weighted proto-feelings. Indeed, the suspension of fast instinctual motor reactions and prototypical every-day attentional attitudes allow more strongly felt sensations (Sarasso et al., 2020a), which, in turn, is *per se* rewarding (Menninghaus et al., 2017). Such aesthetic attitude is usually confined to the artistic domain (Pelowski et al., 2017) [as an example, Gallese, who defines aesthetic distance in terms of freedom, writes: “when watching a film, reading a novel, or beholding a painting, we distance ourselves from the “everyday” context. By adopting such an attitude, our embodied simulation becomes “liberated”—that is, it is freed from the burden of modelling our actual psychophysical presence in daily life; hence, new simulative energies are liberated” (Gallese, 2018; p. 55)]. However, we already discussed that such aesthetic attitude does not necessarily require an artistic context (e.g., being in a museum or attending to a theatre play; Pelowski et al., 2017), but can be triggered by the expectation of beauty itself in the everyday context (Sarasso et al., 2020a). As we demonstrated in a recent paper, the “top-down” expectation of aesthetic pleasure prompted by “bottom-up” aesthetically rewarding experiences, reorients attention from self-referred to environmental stimuli (Sarasso et al., 2022). Similarly, the aesthetic approach in perceiving field forces in therapy is at the root of therapeutic change (Bloom, 2011; Francesetti, 2012). According to this view, change is not about what the therapist does, but it is triggered by the therapist’s aesthetic evaluation of how he/she is “with” the patient (Francesetti, 2015). The expectation of beauty supports the therapist in distancing herself/himself from the desire to change the client and the situation (Francesetti, 2015), a behaviour which, according to the paradoxical theory of change (Beisser, 1970), would prevent full contact with the situation. Similarly to Gallese’s viewpoint, recent approaches in Gestalt therapy describe such attitudinal shift toward aesthetics in terms of freedom: “that shift is generated by curiosity and a feeling of wonder about what is happening” (Bloom, 2009; p. 6). As Husserl’s collaborator Fink (1933) says, “*wonder about the world*” is the best definition of the phenomenological attitude. It is an “enhancement of freedom and a differentiation enabled by a distance from what is seizing us” (Francesetti and Roubal, 2020; p. 4).

### Merging Predictions in the Pre-subjective Chaos

When two or more organisms interact are they governed by separate self-regulation processes, or do they behave as a whole? Do the boundaries of a self-regulating system transcend single organisms? This matter has long been debated with radical and less radical answers. As an example, James Lovelock’s famous Gaia theory hypothesises that the entire planet: “functions in the manner of a vast self-regulating organism, in the context of which all living things collectively define and maintain the conditions conducive for life on earth.” Another illustrious example is the theory of autopoiesis by Humberto Maturana, who dedicated his investigation to the alternative proposition of whole, rather than part, as causal mechanism (Harries-Jones, 2004).

FE minimisation as a self-regulation principle in an ever-changing environment did not solve this ongoing debate. Although most of human’s sensations are caused by other humans, FEP intuitions are insofar mainly limited to the life of single organisms. This is problematic when trying to apply FEP to a phenomenological holistic field theory perspective where subjects and agencies continuously emerge from a common experiential field. The field itself as a whole has its own intentionalities: conservative and transformative forces that precede and transcend individual agencies. FEP accommodates this idea of a decentralised and de-subjectivised (or agent-less, see Friston and Frith, 2015a) intentionality governing field dynamics: we are being moved more than moving. Indeed, according to the more enactive and dynamic readings of the free energy principle: “the brain is not located at the centre of the organism-environment, conducting tests along the radiuses; it’s on the circumference, one station amongst other stations involved in the recursive loop that also navigates through the body and environment and forms the whole. Neural accommodation occurs *via* constant reciprocal interaction between stations: parts are coordinated without an executive agent or programme that produces the organised pattern. Rather, coherence is generated solely in the relationships between the organic components and the constraints and opportunities of the environment. This self-organisation means that no single element has causal priority” (Smith and Thelen, 2003; p. 343). Friston (2013), borrowing Tschacher and Haken (2007) terminology, characterises this reciprocal coupling between an organism and its econiche in terms of “circular causality.” This insight from the FEP has been parallelled to Dewey’s notion of situation (Gallagher and Allen, 2018), which largely inspired field theory in Gestalt therapy. The situation is not equivalent to the environment but includes the agent in such a way that agent and environment are co-defined. When two (or more) agents share and are included in a situation or a field, the field self-organisation might transcend the two, movements are movements of the situations (Gallagher and Allen, 2018).

Furthermore, along these lines, FEP was originally defined as a “mandatory principle” or vitalistic “imperative” that “applies to any biological system […] from single-cell organisms to social networks” (Friston, 2009; p. 293), thus leaving the door open to the application of free energy minimisation dynamics to systems that extends beyond single organisms. Indeed, when investigating shared experiences, such as communication, the author suggests that: “the infinite regress induced by modelling another agent—who is modelling you—can be finessed if you both possess the same model. In other words, the sensations caused by others and oneself are generated by the same process. This leads to a view of communication based upon a narrative that is shared by agents who are exchanging sensory signals. Crucially, this narrative transcends agency” (Friston and Frith, 2015a; p. 1). While interacting, two Bayesian brains do not simply try to predict each other, “they predict themselves” (Friston and Frith, 2015a; p. 1). When two predictive systems are coupled it is possible to assign the hidden states they are trying to infer to both agents and to treat agency as a contextual factor, which transcends individual agency and just contextualises a shared narrative (Friston and Frith, 2015a). Not only action but also perception becomes a shared process when we are together: “What makes social interaction unique, then, is the emergence of this unifying “narrative” (generative model) and its role in shaping our own individualised perception” (Gallagher and Allen, 2018, p. 2640). Sharing a common generative model is a necessary and emergent phenomenon, which might be mathematically described as generalised synchronisation (aka synchronisation of chaotic dynamics; Hu et al., 2010). Generalised synchronisation implies the presence of a *synchronisation manifold*, a set of attractor states that defines the possibilities of the whole coupled system (Friston and Frith, 2015b). Synchronisation between mutual coupled systems and the stability of invariant manifolds (what we somewhere else defined as field forces) are two inseparable phenomena (Brown and Rulkov, 1997; Yamapi et al., 2010). Interestingly, although beyond the scope of the present paper, it has been shown that synchronisation can be used to change the dynamic behaviour of complex systems such as the therapeutic field (Tschacher et al., 2017; Orsucci, 2021).

Altogether, it seems that the free energy principle and predictive coding insights and assumptions can be applied to the therapeutic situation and inform the clinical encounter beyond a mono-personal and bi-personal perspective. Friston’s intuition of a shared generative model, indeed, can explain one of the most controversial aspects of contemporary Gestalt field therapy: the focus on the therapist’s sensation. How to explain the fact that a change in the therapist’s experience of the therapeutic field can produce changes in the patient’s experience as hypothesised by field theory? From a relational or bi-personal perspective this phenomenon can be explained as a sort of contagion (Roubal and Rihacek, 2016). At a different level of explanation, something more complex than simple causality is brought up. From a field perspective, the transformation of a field organisation might start in an undifferentiated pre-subjective dimension, where the proto-feelings push to emerge through the embodiment of client and therapist which primarily works on transforming his/her experience of the field (Francesetti and Roubal, 2020; Roubal and Francesetti, 2022). This intentionality of contacting belongs to the “in between” of the patient-therapist medium, both concurring to maximise the evidence of a shared generative model through actions and perception governed by a common process.

## Conclusion

In the present paper we review the neurocomputational and neurophysiological evidence suggesting that the perception of beauty might have evolved as an epistemic hedonic feedback signalling the reduction of prediction errors over time and the parallel update of the generative model of sensory causes (i.e., the emergence of new Gestalt). In the context of psychotherapy aesthetic sensibility provides a valuable tool to evaluate the match between the direction of therapeutic intervention and the transformative tension of the therapeutic field. Beauty reveals the assimilation of proto-feelings (i.e., prediction errors) into the cognitive and affective model of “what is happening to me.” As any reward, aesthetic pleasure might have evolved to motivate the organism to tolerate a distressing (but profitable in the long-run) situation, such as the experience of sensory uncertainty. This paradoxical search for uncertainty is hedonically marked since it allows humans to learn progressively deeper levels of sensory regularities in the environment. An aesthetic attentional attitude, by supporting the clinician to tolerate transient states of sensory uncertainty, which are felt as disturbing sensory impressions and motor resonances, supports change: proto-feelings that previously lacked the adequate social support to be felt and integrated into experience, can now emerge as proper feelings and affects in the therapeutic encounter. The application of the free energy principle to systems that enclose more than one organism demonstrates the natural emergence of a shared intentionality and a set of attractor states that defines the possibilities of the whole sensory coupled and synchronised system. We discussed how the therapist and the client can be treated as a unique coupled system with (1) its own shared intentionality and (2) a set of attractor states where the organisms involved in the system are more probably found. Hence, the therapeutic field can be treated as a unique metastable organism or system governed by the concurrent tension to maintain a rigid set of states to limit sensory uncertainty and (paradoxically) to encounter sensory uncertainty to maintain a given (predicted) rate of prediction error reduction over time. We hypothesise that beauty in psychotherapy can signal the evolution of attractor states into novel possibilities for experience. More importantly, according to what we propose, focusing on the aesthetic qualities of the therapeutic encounter by maintaining an aesthetic attitude could promote therapeutic change.

Future studies should test the following preliminary empirical predictions driven by our hypothesis:

1) Existence of a moment-by-moment correlation between the emergence of aesthetic emotions subjectively felt by the therapist and the patient during sessions and therapeutic change as evaluated by an independent evaluation group.
2) Presence of a positive correlation between the aesthetic evaluation of the ongoing theraeutic process and subjective reports of therapeutic alliance.
3) Occurrence of a correlation between objective measures of behavioural (and perhaps electrophysiological) synchronisation between the therapist and the patient when the therapist adopts and aesthetic attitude.
4) Better therapeutic outcomes when therapists adopt an aesthetic attitude.
5) The possibility to assess -and train- therapists’ aesthetic competences and interoceptive awareness and to measure the correlation between aesthetic dimensions of training and therapeutic outcomes.

## Author Contributions

PS, GF, JR, and MG developed the manuscript concept. PS and IR drafted the manuscript. KS, JR, GF, and MN-M provided critical revisions. All authors contributed to the review of previous research and approved the final version of the manuscript for submission.

## Conflict of Interest

The authors declare that the research was conducted in the absence of any commercial or financial relationships that could be construed as a potential conflict of interest.

## Publisher’s Note

All claims expressed in this article are solely those of the authors and do not necessarily represent those of their affiliated organizations, or those of the publisher, the editors and the reviewers. Any product that may be evaluated in this article, or claim that may be made by its manufacturer, is not guaranteed or endorsed by the publisher.

## Appendix A

### Glossary

**Gestalt:** Literally a form, pattern or configuration. It is an organisation of the perceptual field that is perceived as more than the sum of its parts.

**Contact:** Subjective experience of the interaction between the organism and the environment, underlying both awareness and motor behaviour (Perls et al., 1951; p. 227).

**Support:** Everything that facilitates the ongoing assimilation and integration of experience for a person, relationship, or society (Perls, 1992; p. 132).

**Growth:** The function of the contact between the organism and the environment (Perls et al., 1951; p. 230): the assimilation of sensory novelty.

**Intentionality of contacting (contact intentionality):** The emerging tension towards the contact between the therapist and the client, which moves the interaction towards the actualisations of the potentialities in the hic et nunc of the therapeutic situation (Roubal et al., 2017).

**Enactive:** The property of the dynamic interaction between an organisms and its environment for which the environment is brought about, or enacted, by the active exercise of that organism’s sensorimotor processes.

**Field of experience:** The emergent relational phenomenon transcending subjects, perceptible by the senses and sensori-motor resonances, in which certain experiential phenomena tend to emerge, while others do not.

**Markov blanket:** A mathematical description that defines the boundaries between the organism (e.g., a cell or a multi-cellular organism) and the environment in a statistical sense, by partitioning into internal and external states. External states are conditionally independent of internal states, and vice versa, as internal and external states can only influence each other *via* the blanket states.

**Ergodicity:** The property of an organism which occupies or revisits over time some typical states more than others over time in order to maintain its structural and dynamical integrity.

**Econiche:** A limited set of states of the world occupied by a given organism.

**Generative model:** The living being must actively predict which set of states it will probabilistically encounter by creating representations of environmental and internal dynamics. Such representations are in fact probability distributions, or what the author calls “generative models” (Friston et al., 2006). The model that better explains sensory input is selected and consciously experienced. It continuously adapts to account for novel stimuli. When the agent gathers new sensory evidence, it must combine a likelihood function with its prior beliefs.

**Autopoiesis:** An autopoietic system (Varela, 1997) is organised as a network of processes of production (synthesis and destruction) of molecules such that the system: (1) continuously regenerate and realise the network that produces the molecules, (2) constitutes as a distinguishable unity in the domain in which it exists, and (3) is able to potentially distinguish the different virtual implications of otherwise equally viable paths of encounters with the environment.

**Enactive:** Enactive theories posit that cognition does not passively receive information from their environments, which they then translate into internal representations. Organisms participate in the generation of meaning by the active exercise of that organism’s sensorimotor processes.

**Metastability:** Fom latin, *meta* (beyond), *stabilis* (stable): the simultaneous realisation of two competing tendencies of parts of complex self-regulating systems to converge on and destroy stable states and create new dynamics.
